# Dietary Supplementation of *Chlorella vulgaris* Effectively Enhanced the Intestinal Antioxidant Capacity and Immune Status of *Micropterus salmoides*

**DOI:** 10.3390/antiox12081565

**Published:** 2023-08-04

**Authors:** Heng Yu, Xianping Ge, Dongyu Huang, Chunyu Xue, Mingchun Ren, Hualiang Liang

**Affiliations:** 1 Wuxi Fisheries College, Nanjing Agricultural University, Wuxi 214081, China; 2020213004@stu.njau.edu.cn (H.Y.); gexp@ffrc.cn (X.G.); 2022213004@stu.njau.edu.cn (C.X.); 2 Key Laboratory of Integrated Rice-Fish Farming Ecology, Ministry of Agriculture and Rural Affairs, Freshwater Fisheries Research Center, Chinese Academy of Fishery Sciences, Wuxi 214081, China; huangdongyu1995@163.com

**Keywords:** *Chlorella vulgaris* (*C. vulgaris*, CHL), *Micropterus salmoides* (*M. salmoides*), transcriptome analysis, antioxidant capacity, immunity

## Abstract

An *M. salmoides* fish meal diet was supplemented with 0 (CHL0, Control), 38 (CHL38), 76 (CHL76), 114 (CHL114), and 152 (CHL152) mg/kg *C. vulgaris* for 60 days, and their serum and intestinal samples were analyzed. The results showed that the albumin (ALB) and total protein (TP) contents were observably enhanced in the CHL76 group compared with the Control group. The intestinal glutathione (GSH) and glutathione peroxidase (GSH-Px) contents were enhanced significantly in the CHL76 group, while the total antioxidant capacity (T-AOC) was enhanced in the CHL38 group, compared with the Control group. However, supplementation of >76 g/kg *C. vulgaris* significantly inhibited the superoxide dismutase (SOD) activity in the intestines of *M. salmoides*. Moreover, the malondialdehyde (MDA) content was observably dropped in the CHL-supplemented groups compared with the Control group. Transcriptome analysis of the CHL76 and Control groups displayed a total of 1384 differentially expressed genes (DEGs). KEGG analysis revealed that these DEGs were enriched in apoptosis, cytokine–cytokine receptor interaction, tight junction (TJ), and phagosome signaling pathways, which were associated with improved intestinal immunity in the CHL76 group. Additionally, the DEGs enriched in the above pathways were also correlated with the antioxidant parameters, such as catalase (CAT), GSH, GSH-Px, SOD, T-AOC, and MDA. Therefore, our study found that dietary supplementation of *C. vulgaris* effectively enhanced the intestinal antioxidant capacity of *M. salmoides* by increasing antioxidant enzyme activity and decreasing MDA content. Additionally, dietary supplementation of *C. vulgaris* improved the intestinal immune status of *M. salmoides* by reducing proapoptotic and proinflammatory factors, increasing intestinal TJs- and phagosome-related genes expressions, and increasing the serum ALB and TP contents. Lastly, quadratic regression analysis of the serum biochemical indices (ALB and TP) and intestinal antioxidant parameters (GSH-Px and GSH) revealed that the optimal supplemental level of *C. vulgaris* in the *M. salmoides* diet was 58.25–77.7 g/kg.

## 1. Introduction

Aquaculture contributes to global food security by providing high-quality protein [1], and according to the Food and Agriculture Organization (FAO) [2], fish accounted for approximately 17% of global animal protein consumption in 2017. In 2020, freshwater fish was the most prominent biota in aquaculture, with fin fish farming reaching 8.3 million tons in seawater and 49.1 million tons in inland aquaculture [3], thus indicating the importance of freshwater fish farming in global food production. *Micropterus salmoides* (*M. salmoides*) is a widely farmed freshwater fish that first originated in North America and was then introduced in various countries, including China, Japan, and New Zealand. *M. salmoides* is preferred by both fish farmers and consumers owing to its several favorable characteristics, such as rapid growth, adaptability, lack of thorns, flavorful meat, etc. [4]. *M. salmoides* is a carnivorous fish [5], and the demand for fish meal (FM) is 40–50% [6]. According to the FAO [7], compared to 2018, the yearly output of FM in 2030 would rise by 1% while its price would increase by 30%. This would not only increase the cost of feeding but also lead to restrictions on the farming industry in the future. Therefore, finding novel protein sources that can fully or partially replace FM can promote sustainable development of the aquaculture industry and contribute to the global production of high-quality aquatic animal protein.

The alternative protein sources for FM can be classified as animal, plant, or single-cell protein (SCP) sources. Among the SCPs, microalgae, bacteria, and yeast have attracted the attention of researchers due to their several advantages. For instance, compared to terrestrial plants, microalgae grow more rapidly and produce more biomass owing to their higher efficiency in harnessing sunlight and CO_2_ [8]. *Chlorella vulgaris* (*C. vulgaris*, CHL), a freshwater microalga, is known for its easy cultivation and high yield [9]. Moreover, it is nutrient-rich and does not compete with humans for land or food [10]. Additionally, *C. vulgaris* contains *Chlorella* growth factor (CGF), which is rich in nutrients, such as amino acids, peptides, and polysaccharides [11]. In Pacific white shrimp (*Litopenaeus vannamei*), *C. vulgaris* could replace 100% of the FM without causing any adverse effects due to the presence of antioxidants, minerals, vitamins, and other nutrients [12]. *C. vulgaris* has been used to partially substitute FM for Nile tilapia (*Oreochromis niloticus*) [13], Gilted seabream (*Sparus aurata*) [14], and narrow clawed crayfish (*Pontastacus leptodactylus Eschscholtz*) [15]. In our previous study, we found that *C. vulgaris* could effectively replace 15.56% of the FM in the *M. salmoides* diet [16].

When the dysfunction of the intestinal epithelial barrier may impair intestinal function, the intestinal tract would select some pathogenic bacteria or harmful organisms to enter the intestinal tract, thus endangering intestinal health [17]. Existing studies focus on the effect of dietary supplementation of *C. vulgaris* on the growth, intestinal health, and intestinal microbiota of *M. salmoides* [18,19,20]. However, few studies have been conducted on how *C. vulgaris* regulates the immune and antioxidant mechanisms of the intestine, and transcriptome sequencing provides a good technique for explaining its mechanism. Therefore, in the current research, we determined the effects of the partial substitution of FM by *C. vulgaris* on the antioxidant and immune functions of *M. salmoides* and explored its potential mechanism using transcriptome analysis.

## 2. Materials and Methods

### 2.1. Experimental Design

All the subjects of the current study, *M. salmoides*, were provided by Yongda Aquaculture Professional Cooperative (Ezhou, China). The experiments were carried out in floating cages in an earthen pond at Wuhan Cp Aquatic Co., Ltd. (Huanggang, China). Prior to experimentation, *M. salmoides* were kept in net cages (1 × 1 × 1 m) for 14 d to acclimatize them to the culture conditions. In the formal experiment, a total of five experimental groups were designed, with three replicates in each group and 20 fish (15 ± 0.2 g) in each replicate. Fish were hand-fed to apparent satiation twice a day for 60 days. The *M. salmoides* were fed daily at 6:30 am and 6:30 pm, following the principle of full feeding. Water temperature ranged from 28 °C to 31 °C, dissolved oxygen was maintained above 6.0 mg/L, pH fluctuated between 7.5 and 8.2, ammonia nitrogen and nitrite ranged between 0–0.2 mg/L and 0.1–0.3 mg/L, respectively, during the feeding trial.

### 2.2. Experimental Diets Preparation

*C. vulgaris*, containing 60.83% protein and 10.40% lipid, was obtained from Wuxi Tongwei Feedstuffs Co., Ltd. (Wuxi, China). The five isonitrogenous and isoenergetic diets were formulated to contain 0 (CHL0), 38 (CHL38), 76 (CHL76), 114 (CHL114), and 152 (CHL152) mg/kg *C. vulgaris*. The experimental feed composition is provided in our previously published article [16]. The main protein raw material used in current research were all obtained by crushing them with a grinder and then sieving them pass a 60-mesh sieve and mixed sequentially with water and fish oil. Thereafter, the protein samples were processed with an F-26 (II)-type granulator to make the feed, which was then air-dried, placed in a self-sealing bag, and stored at −20 °C.

### 2.3. Fish Sample Collection

The fish were fasted 24 h prior to sampling. After anesthesia with MS-222 (100 mg/L), nine fish were randomly selected from each group (three per replicate) for serum biochemical index analysis. The collected blood was centrifuged to obtain serum (3500× *g*, 10 min, 4 °C), which was then stored at −80 °C for analysis of serum biochemical indices. Additionally, six fish were randomly selected from each group (two per replicate) to analyze the intestinal antioxidant indices. Lastly, three fish each were selected from the Control and CHL76 groups (1 per replicate) for transcriptome analysis. All the tissue samples were placed into frozen storage tubes and immediately placed in a liquid nitrogen tank. The frozen samples were transferred to a −80 °C freezer until further analysis.

### 2.4. Analysis of the Serum Biochemical Indices and Intestinal Antioxidant Parameters

The assay kits or testing equipment for the detection of the serum biochemical indices and the intestinal antioxidant parameters are shown in Table 1.

### 2.5. The Process of High-Throughput Sequencing

According to its instructions, TRIzol was used to extract intestinal RNA. The purity and concentration of RNA were detected using a NanoDrop 2000 spectrophotometer (Thermo Scientific) (New York, NY, USA) and the integrity of the RNA was accurately detected via Agient2100/LabChip GX (Perkin Elmer LabChip GX) (New York, NY, USA) to ensure the quality of the RNA. The RNA samples were used for library construction and quantified using a Qubit 3.0 fluorescence quantizer (Thermo Fisher Scientific) (New York, NY, USA) (>1 ng/μL). Thereafter, the Qsep400 high-throughput analysis system was used to assess the inserted fragments of the library. The effective concentration of the library needs quantitative real-time PCR (qPCR) assay to quantify accurately and to control the quality of the library so that it can be further used. Lastly, the Illumina NovaSeq6000 sequencing platform was used in the PE150 mode for library sequencing.

### 2.6. Quality Inspection of Sequencing Data

Before data analysis, remove the Reads containing joints and low-quality Reads. These Reads must be of high quality for the accuracy of further analysis. The positioning information on the reference genome was obtained by quickly and accurately comparing Clean Reads with the reference genome using the HISAT (2.0) software [21], and the aligned sequences were assembled using StringTie to reconstruct the transcriptome for subsequent analysis [22]. Based on the count value of genes in each sample, DESeq2 [23] was used to screen DEGs at fold change ≥2 and a false discovery rate < 0.05.

### 2.7. Quantitative Real-Time PCR Detection

RNA was extracted (on ice) from the *M. salmoides* intestinal samples using an RNA extraction reagent (Vazyme, Nanjing, China). The RNA concentration and quality were measured using a NanoDrop 2000 spectrophotometer, and RNA samples with A260/A280 of 1.8–2.0 were used for qPCR assay. The qPCR assay was conducted on the CFX96 Touch (Bio-Rad, Hercules, CA, USA) using reagents obtained from Vazyme. A standard curve was used to calculate the mRNA levels, and *β-actin* was proven to be usable in *M. salmoides*, which can act as a housekeeping gene. The gene sequences are shown in Table 2.

### 2.8. Statistical Analysis

Data were analyzed via one-way analysis of variance (ANOVA) using SPSS (20.0) and Tukey’s test for pairwise comparison (*p* < 0.05). Suppose the comparison between the two groups was the result of analysis by independent sample *t*-test. The data are presented as mean ± standard error.

## 3. Results

### 3.1. Effects of Dietary Supplementation of C. vulgaris on the Serum Biochemical Indices

As seen in Table 3, the ALB and TP levels were significantly increased in the CHL76 group compared to the Control group (*p* < 0.05). The differences in ALT and AST contents among *M. salmoides* fed different *C. vulgaris* levels were insignificant (*p* > 0.05).

### 3.2. Effects of Dietary Supplementation of C. vulgaris on the Intestinal Antioxidant Parameters

**Figure 1:**
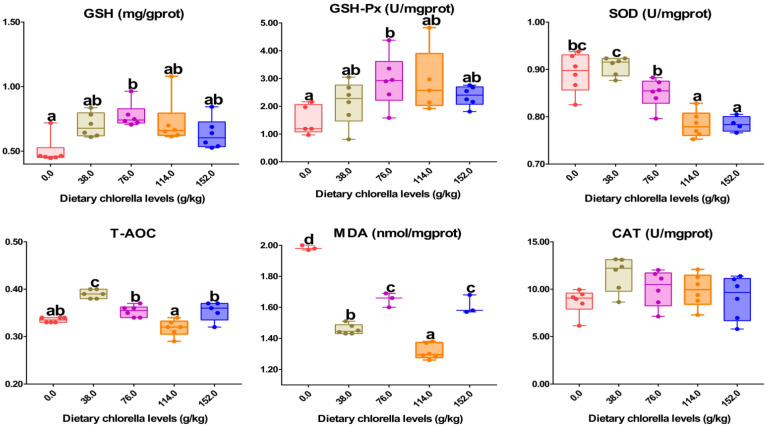
Glutathione (GSH), glutathione peroxidase (GSH-Px), superoxide dismutase (SOD), total antioxidant capacity (T-AOC), malondialdehyde (MDA), and catalase (CAT). Data are expressed as means with standard error. Values with different alphabetical superscripts were significantly different (*p* < 0.05).

As seen in Figure 1, the intestinal GSH content of *M. salmoides* fed CHL76 and CHL114 diets was observably raised in comparison with the Control group (*p* < 0.05). The GSH-Px content showed an upward trend, with a significant increase in the CHL76 group (*p* < 0.05). The intestine SOD activity and T-AOC of *M. salmoides* were significantly increased in the CHL38 group in comparison with the Control group (*p* < 0.05). Nevertheless, MDA levels decreased in the CHL-supplemented groups (*p* < 0.05). Insignificant differences were found in intestinal CAT activity among *M. salmoides* fed different *C. vulgaris* levels (*p* > 0.05).

### 3.3. Optimal Supplementation Level of C. vulgaris in M. salmoides Diet

**Figure 2:**
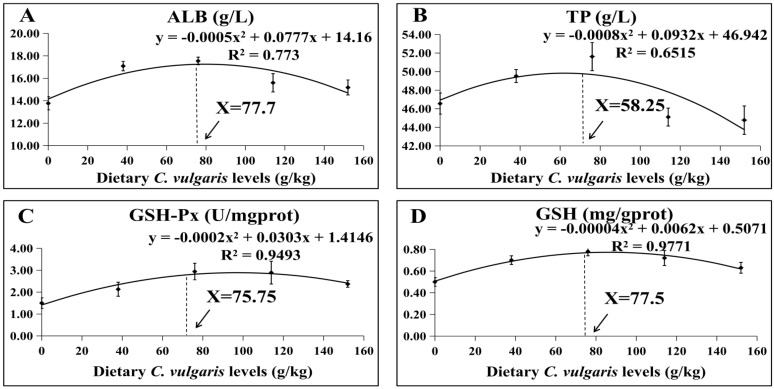
Albumin (ALB) (**A**), total protein (TP) (**B**), glutathione peroxidase (GSH-Px) (**C**), and glutathione (GSH) (**D**). The data were described in terms of mean ± standard error.

As seen in Figure 2, Quadratic regression analysis of ALB, TP, GSH-Px, and GSH revealed that the appropriate supplementation amount of *C. vulgaris* in the *M. salmoides* diet was 77.7, 58.25, 75.75, and 77.5 g/kg, respectively.

### 3.4. Library Sequencing Quality

As seen in Table 4, each sample produced ≥38,524,894 total Reads, and the average Clean Reads of the Control and the CHL-supplemented groups were >42,939,487 and >43,576,963, respectively. Their GC content was 47.26–49.08%, and the proportion of bases with mass value ≥30 was ≥93.62% in all the samples. Altogether, these results indicate that the library sequencing quality met the standards required for DEG analysis.

### 3.5. DEG Analysis

**Figure 3:**
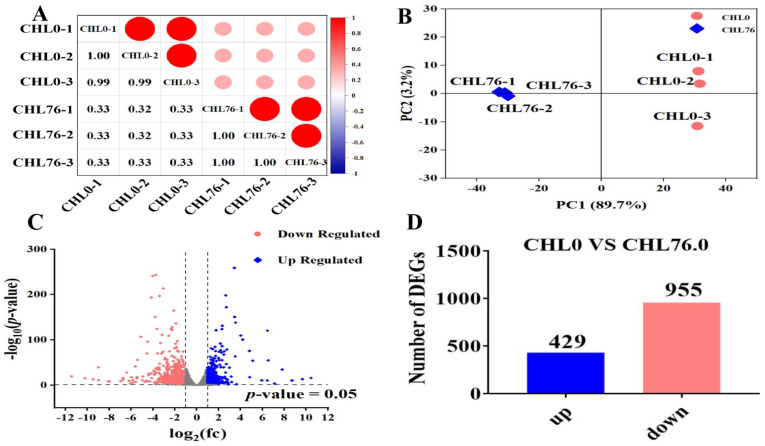
Correlation analysis between samples (**A**), the circle size indicated a correlation between samples (the larger the size, the stronger the correlation); The PCA diagram shows the distribution of data in the principal component space (**B**); The bubble map shows the upregulated and downregulated DEGs (**C**); Total DEGs in *M. salmoides* fed CHL76 in comparison with the control diet (**D**).

Correlation analysis between samples (Figure 3A) and the PCA diagram (Figure 3B) were used as measures of differences between samples within and between groups. The closer the r^2^ value is to 1, the better the repeatability between the samples, and vice versa (Figure 3A). In the current research, the PCA diagram displayed that the three samples in the same group were clustered, indicating lower within-group differences but higher between-group differences. It was observed that the intra-group samples used in the current research were more reproducible and had a good degree of discrimination between the groups. A total of 1384 DEGs were identified between the CHL76 and Control groups (Figure 3C), and among these, 429 DEGs were observably upregulated, while 955 DEGs were observably downregulated in the CHL76 group (Figure 3D). The KEGG pathway enrichment analysis of the DEGs showed that 1153 DEGs were enriched in 167 signaling pathways, among which most DEGs were enriched in immune- and antioxidant-related pathways. Additional information on the inflammation-related DEGs is shown in Table 5.

### 3.6. qPCR Assay

**Figure 4:**
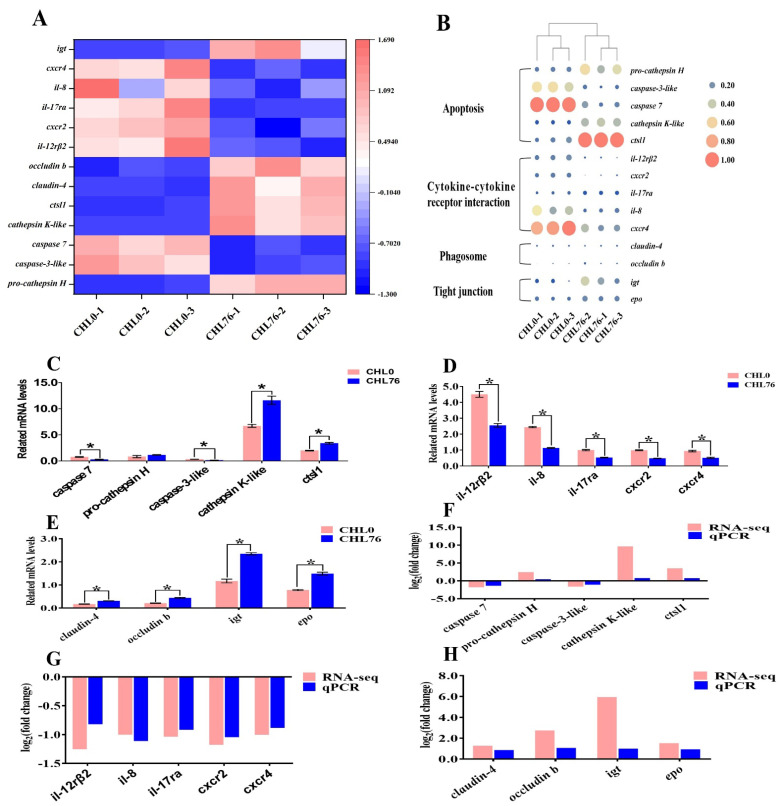
The visual heatmap between pro-cathepsin H, caspase-3-like, caspase-7, cathepsin K-like, cathepsin L1 (*ctsl1*), interleukin-12 receptor subunit beta-2 (*il-12rβ2*), chemokine (C-X-C motif) receptor 2 (*cxcr2*), interleukin-17 receptor A-like (*il-17ra*), interlukin-8 (*il-8*), C-C motif chemokine 4-like (*cxcr4*), claudin-4, occludin b, eosinophil peroxidase (*epo*), and immunoglobulin tau heavy chain (*igt*) in RNA-seq results and samples (**A**,**B**). qPCR is used to verify the validity of DEGs (**C**–**H**). The data were described in terms of mean ± standard error. The asterisk indicates a significant difference between the two groups (*p* < 0.05).

As described in Figure 4, the heat map between DEGs and samples in transcriptome results (Figure 4A,B). The high-throughput sequencing results indicated that the genes pro-cathepsin H, cathepsin K-like, cathepsin L1 (*ctsl1*), occludin b, claudin-4, eosinophil peroxidase (*epo*), and immunoglobulin tau heavy chain (*igt*) were significantly upregulated, while caspase-7, caspase-3-like, C-C motif chemokine 4-like (*cxcr4*), interleukin-8 (*il*-8), il-17 receptor A-like (*il-17ra*), chemokine (C-X-C motif) receptor 2 (*cxcr2*), and il-12 receptor subunit beta-2 (*il*-*12rβ2*) were observably downregulated in the CHL76 group compared with the Control group (Figure 4C–H). These were consistent with the results of RNA-seq and qPCR assays, thus indicating their credibility.

### 3.7. Correlation Analysis between DEGs, Serum Biochemical Indices, and Antioxidant Parameters

**Figure 5:**
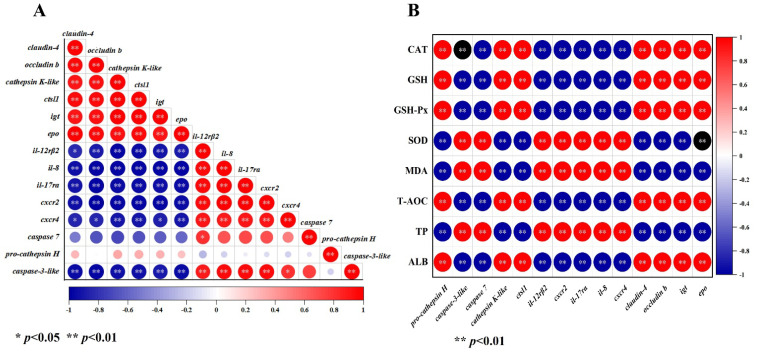
The correlation analysis among DEGs (**A**) and the correlation analysis between DEGs and serum biochemical indexes and antioxidant parameters (**B**). The asterisk indicates a significant difference between the two indicators (* *p* < 0.05, ** *p* < 0.01). Cathepsin L1 (*ctsl1*), interleukin-12 receptor subunit beta-2 (*il-12rβ2*), chemokine (C-X-C motif) receptor 2 (*cxcr2*), interleukin-17 receptor A-like (*il*-*17ra*), interlukin-8 (*il*-*8*), C-C motif chemokine 4-like (*cxcr4*), eosinophil peroxidase (*epo*), immunoglobulin tau heavy chain (*igt*). Glutathione (GSH), glutathione peroxidase (GSH-Px), superoxide dismutase (SOD), total antioxidant capacity (T-AOC), malondialdehyde (MDA), catalase (CAT), albumin (ALB), and total protein (TP).

As shown in Figure 5, visualized heat maps of the correlation between DEGs (Figure 5A) and the correlation between DEGs and antioxidant parameters were analyzed (Figure 5B). This showed that the *caspase-3-like* and cytokine–cytokine receptor interaction-related DEGs (*il-12rβ2*, *il-8*, *il-17ra*, *cxcr2*, and *cxcr4*) were negatively associated with the tight junction protein-related DEGs (*occludin b* and *claudin-4*), apoptosis-related DEGs (*cathepsin K-like* and *ctsl1*), and phagosome-related DEGs (*igt* and *epo*). However, *caspase-3-like* was positively correlated with the cytokine–cytokine receptor interaction-related DEGs (*il-12rβ2*, *il-8*, *il-17ra*, *cxcr2*, and *cxcr4*). Moreover, there was no significant correlation between *pro-cathepsin H* and other DEGs, while *caspase-7* was positively correlated with only *il-12rβ2*. Altogether, these results indicate that all the DEGs were associated with antioxidant parameters. The ALB, CAT, GSH-Px, T-AOC, and GSH were positively associated with the TJ-related DEGs (*occludin b* and *claudin-4*), apoptosis-related DEGs (*cathepsin K-like*, *ctsl1*, and *pro-cathepsin H*), and phagosome-related DEGs (*igt* and *epo*). However, CAT, GSH-Px, T-AOC, and GSH were negatively correlated, while MDA was positively correlated with cytokine–cytokine receptor interaction-related DEGs (*il-12rβ2*, *il-8*, *il-17ra*, *cxcr2*, and *cxcr4*) and apoptosis-related DEGs (*caspase-7* and *caspase-3-like*).

## 4. Discussion

Animals have a complex antioxidant system, including antioxidant enzymes and non-enzymatic antioxidants [24], and *C. vulgaris* supplementation was found to effectively improve the antioxidant capacity of animals, such as rabbits [25]. In the current research, the T-AOC was observably enhanced in the CHL38 group compared with the Control group, while the MDA decreased significantly with CHL supplementation. The current results were similar to the previous study on Grey mullet (*Mugil cephalus*), which found that CHL supplementation could significantly increase the T-AOC level and significantly decrease the MDA level [26]. In general, the MDA level indicates the degree of lipid peroxidation in the body, thus indirectly indicating the degree of cell damage [27]. A study found that *C. vulgaris* has a better inhibitory effect on lipid peroxidation compared to glutathione, suggesting that it has antioxidant properties [28]. In this study, we found that the GSH content and GSH-Px activity increased significantly in the CHL76 group compared with the Control group, indicating that dietary supplementation of *C. vulgaris* can improve the intestinal antioxidant capacity of *M. salmoides* by increasing the levels of antioxidant indices and reducing the MDA level. The antioxidant properties of *C. vulgaris* may be due to the presence of several antioxidant bioactive factors [29]. For example, *C. vulgaris* contains abundant astaxanthin, a type of carotene, which is known as super vitamin E due to its natural antioxidant activity [30]. A previous study found that dietary supplementation of astaxanthin can improve the antioxidant and immune capacity of Rainbow trout (*Oncorhynchus mykiss*) [31]. Moreover, Ghwenm et al. reported that the ethanol extract of *C. vulgaris* contains antioxidant substances that can resist oxidative stress [32]. Serum biochemical parameters are commonly used as indicators of the health status of a fish [33]. In this study, we found no significant differences in the serum AST and ALT contents between the Control and CHL-supplemented groups, which indicates that dietary supplementation of *C. vulgaris* does not cause damage to *M. salmoides*. Additionally, the present results showed that the CHL76 group significantly increased TP and ALB activities. In general, increased TP and ALB levels may be an important parameter of the immune status of the fish [34]; therefore, our results indicate that CHL supplementation can improve the immune status of *M. salmoides*. Altogether, these results suggest that dietary supplementation of *C. vulgaris* improves the antioxidant capacity and immune status of *M. salmoides*. The quadratic regression analysis of the serum biochemical indices (ALB and TP) and intestinal antioxidant parameters (GSH-Px and GSH) revealed that 58.25–77.7 g/kg was the optimal supplemental level of *C. vulgaris* in *M. salmoides* diet.

In this study, we used transcriptome analysis to further examine the underlying molecular regulatory mechanisms associated with improved antioxidant capacity and immune status of *M. salmoides*. We found a total of 1384 DEGs between the CHL76 and Control groups, and KEGG enrichment analysis of these DEGs revealed that some immune-related DEGs were enriched in the apoptosis, cytokine–cytokine receptor interaction, TJ, and phagosome signaling pathways. Apoptosis is mediated by the activation of apoptotic factors by proteolytic enzymes called caspases, a family of cysteine-specific proteases that occupy an important position in the immune response against pathogens [35]. The *caspase-3-like* proteases play an important role under stress conditions [36], and an increase in *caspase-3-like* protease activity indicates the occurrence of cell apoptosis [37,38]. *Caspase-7*, a major molecule involved in cell apoptosis, can be induced by pathogenic infections and has been found in fish [39,40]. In the current study, we found that the expression of intestinal *caspase-3-like* and *caspase-7* genes was significantly downregulated in the CHL-supplemented. A previous study found that dietary supplementation of *methanotroph bacteria* and Yellow mealworm (*Tenebrio Molitor*) could reduce intestinal pro-apoptotic factors and enhance intestinal immunity of *M. salmoides* [41,42]. This showed that dietary supplementation of *C. vulgaris* downregulated intestinal pro-apoptotic factors in *M. salmoides*. Müller et al. found that cysteine *ctsl1* can activate macrophages to eliminate *Staphylococcus aureus* through the production of bactericidal peptides or proteins [43]. Additionally, existing results have found that *ctsl1* occupies a vital position in the immunity of freshwater mussels (*Cristaria plicata*) [44] and pacific abalone (*Haliotis discus hannai*) [45]. Moreover, the *ctsl1* and *cathepsin K-like* genes were downregulated under external stress in Nile tilapia [46]. In the current study, the apoptosis pathway-enriched genes, *cathepsin K-like*, *ctsl1*, and *pro-cathepsin H*, were significantly upregulated in the CHL-supplemented groups. *Cathepsin* is a widely distributed protease in both prokaryotes and eukaryotes, and it plays important immune functions, including lysosome protein degradation, in fishes and other vertebrates [47,48,49]. Altogether, these results indicate that dietary supplementation of *C. vulgaris* significantly improves the immune status of *M. salmoides*. The activation of the immune system requires the consumption of energy, and this energy consumption may be worth consuming for improving the health of fish, as the improvement of immunity is considered to be one of the evaluation criteria for the good application effect of *C. vulgaris* in fish. When the immune system of fish was activated, how much energy would be consumed and whether the energy consumed would affect the growth and health of fish was very meaningful to explore.

Intestines serve as a key immune and physical barrier to prevent the entry of harmful substances [50]. In fish, *IL-8* acts as a proinflammatory factor [51]. Moreover, the expression of the *il-17ra* gene increases significantly in the fish intestines under external stress [52], while the expression of *il-12rβ2* increases significantly in the fish intestines after *Aeromonas hydrophila* infection [53,54]. *CXCR2* is mainly expressed in inflammatory cells [55] and can be activated by several chemokines, including *IL-8* [56,57,58]. Like most chemokine receptors, *CXCR4* contributes to inflammatory diseases and cancers [59,60]. The results of our study revealed that all these genes were differentially expressed and were enriched in the cytokine–cytokine receptor interaction pathway, whose dysfunction is associated with pathological alterations in the body, including increased inflammation [61,62]. In the current research, proinflammatory genes, including *il-12rβ2*, *cxcr2*, *il-17ra*, *il-8*, and *cxcr4*, were significantly downregulated in the CHL76 group, indicating that *C. vulgaris* has anti-inflammatory effects. Moreover, a previous report concluded that proinflammatory factors can inhibit TJ protein genes expressions observably [63].

TJ proteins are closely associated with the structural integrity of the intestines, and the increase in TJ (*occ-1b* and *occludin*)-related gene expressions in the intestine represents an improvement in intestinal health [64,65], while a decrease in the expression of *claudin-4* indicates the loss of intestinal barrier function [66]. In the current research, the expression of *occludin b* and *claudin-4* was observably enhanced in the CHL-supplemented groups compared with the Control group. This is similar to a previous study that found that *claudin-4* expression increased significantly in the CHL-supplemented *M. salmoides* group compared with the Control [19]. *EPO* has antibacterial activity and occupies a crucial position in intestinal immunity [67]. Previous reports have suggested that *IgT* may play a crucial role in intestinal and gill mucosal immunity of *M. salmoides* [68] and rainbow trout [69,70]. In the current research, the expression of *igt* and *epo*, enriched in the phagosome signaling pathway, was observably increased in the CHL76 group compared with the Control group. This revealed that dietary supplementation of *C. vulgaris* can strengthen the intestinal barrier and improve the intestinal health of *M. salmoides*.

KEGG enrichment analysis revealed that the DEGs were closely associated with immunity and that the DEGs were correlated with serum biochemical index, ALB, and antioxidant parameters, such as CAT, GSH, GSH-Px, SOD, T-AOC, and MDA. Moreover, ALB, CAT, GSH, GSH-Px, and T-AOC were positively correlated, while MDA was negatively correlated with *cathepsin K-like*, *ctsl1*, *pro-cathepsin H*, *claudin-4*, *occludin b*, *igt*, and *epo*. The increase in ALB, CAT, GSH-Px, T-AOC, and GSH is an important indicator of the improvement in the immune and antioxidant capacity of an organism, while an increase in MDA indicates the occurrence of oxidative damage. These results indicate a correlation between antioxidant capacity and immune factors; however, further research is required to determine the specific mechanisms by which immune factors interact with antioxidant indicators.

## 5. Conclusions

**Figure 6:**
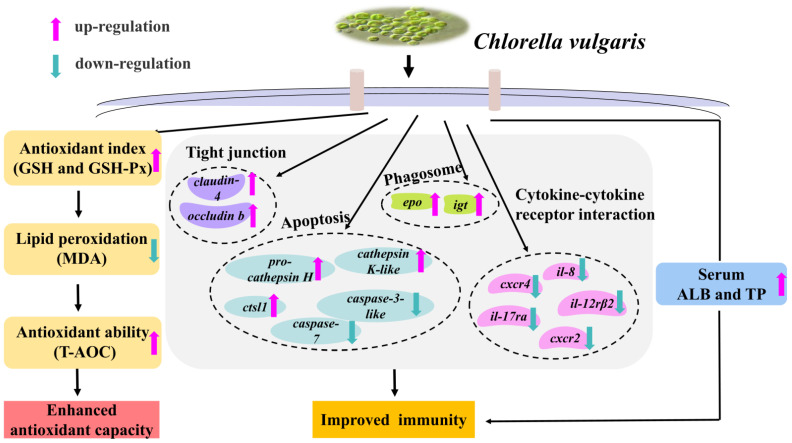
The potential regulatory hanisms for enhancing antioxidant capacity and improving immunity in juvenile *M. salmoides* fed with *C. vulgaris*. *Cathepsin L1* (*ctsl1*), *interleukin-12 receptor subunit beta-2* (*il-12rβ2*), *chemokine (C-X-C motif) receptor 2* (*cxcr2*), *interleukin-17 receptor A-like* (*il-17ra*), *interlukin-8* (*il-8*), *C-C motif chemokine 4-like* (*cxcr4*), *eosinophil peroxidase* (*epo*), *immunoglobulin tau heavy chain* (*igt*). Glutathione (GSH), glutathione peroxidase (GSH-Px) total antioxidant capacity (T-AOC), malondialdehyde (MDA),albumin (ALB), and total protein (TP).

The dietary supplementation of *C. vulgaris* effectively enhanced the intestinal antioxidant capacity of *M. salmoides* by increasing the activities of antioxidant enzymes and decreasing the MDA content. Furthermore, dietary supplementation of *C. vulgaris* improved the intestinal health of *M. salmoides* by reducing the expression of proapoptotic and proinflammatory factors, increasing the expression of intestinal TJ- and phagosome-related genes, and increasing the serum ALB and TP contents (Figure 6). Lastly, quadratic regression analysis of the serum biochemical indices (ALB and TP) and intestinal antioxidant parameters (GSH-Px, and GSH) revealed that 58.25–77.7 g/kg was the optimal supplemental level of *C. vulgaris* in the *M. salmoides* diet.

## Figures and Tables

**Table 1:** The assay kits or testing equipment of serum biochemical indices and intestinal antioxidant parameters.

Items	Methods/Assay Kits/Testing Equipment
ALT	The serum biochemical indices were measured using an automatic biochemical analyzer, a Mindary BS-400 (Shenzhen, China) and assay kits (Mindray) purchased from Gansu Heyuan Biotechnology Co., Ltd. (Gansu, China).
AST	
ALB	
TP	
GSH	The intestinal antioxidant parameters were detected using the kits purchased from Nanjing Jiancheng Bioengineering Institute (Nanjing, China), and the samples were prepared according to the manufacturer’s instructions.
GSH-Px	
SOD	
T-AOC	
CAT	
MDA	

Note: Alanine aminotransferase (ALT), aspartate aminotransferase (AST), albumin (ALB), and total protein (TP), glutathione (GSH), glutathione peroxidase (GSH-Px), superoxide dismutase (SOD), total antioxidant capacity (T-AOC), malondialdehyde (MDA), and catalase (CAT).

**Table 2:** Primer sequences for quantitative real-time PCR assay.

Gene Name		Sequence	Tm (°C)	GC (%)	Accession No.
*pro-cathepsin H*	F	AAGGCCATGGTTGATGCTGT	60.25	50	XM_038701870.1
	R	CAGACTGTGCCCCAAGAGTT	59.89	55	
*caspase-3-like*	F	CCCTCAGCAAACTGGGCTT	60.23	57.89	XM_038739762.1
	R	TGAGGAACACTTTGGCCTTTTTC	59.87	43.48	
*caspase 7*	F	CCTACACCTTCCAGGCCAAA	59.6	55	XM_038734228.1
	R	CGCAGACATCAGTTGCTCAC	59.56	55	
*cathepsin K-like*	F	AGGGCCATTTGGGAGAAGAAC	60.27	52.38	XM_038694428.1
	R	CGATTTGGGAAGCTTGGACAC	59.8	52.38	
*ctsl1*	F	AGATCGAGCTGCACAACCTG	60.39	55	XM_038704447.1
	R	CACTGACCCTGGTCCTTCAC	59.96	60	
*il-12rβ2*	F	TCCAGTATCGGACTGAGGCA	60.03	55	XM_038698864.1
	R	TCGAAGCTTGCAGGGAATGT	59.96	50	
*cxcr2*	F	CAGGTTGGACATAGTGCCGT	60.04	55	XM_038721015.1
	R	AAGACCTGCTGCTTCTTGCT	59.89	50	
*il-17ra*	F	ATGTGTGGCGACAAAGAGGT	59.89	50	
	R	GTGATTCACTCTGCCCGGTT	60.32	55	
*il-8*	F	GAGGGTACATGTCTGGGGGA	60.33	60	XM_038713529.1
	R	CCTTGAAGGTTTGTTCTTCATCGT	59.72	41.67	
*cxcr4*	F	GGTCCAGATGACTGCTGCTT	60.04	55	XM_038726066.1
	R	GCTGGATCACTCGGATGGTT	59.82	55	
*claudin-4*	F	TGAGGTACTCCAAGGCTCGT	60.25	55	XM_038707645.1
	R	GCAACAATGGTGTAGGGGGA	59.96	55	
*occludin b*	F	GGTCTGGGAAGTGGAGTTGG	59.96	60	XM_038703759.1
	R	TGGTGAGCGGGCAGTATTTT	59.96	50	
*igt*	F	CTTCTGCTGGTTGCTCTCTCT	59.72	52.38	
	R	GCTGGCGTAATCTGTTTTGCT	59.8	47.62	
*epo*	F	ATCTCGGCAGTCCTCTCCTT	60.03	55	XM_038733743.1
	R	TTGCGTTGAGTGAGCGTTTG	59.97	50	

Note: cathepsin L1 (*ctsl1*), interleukin-12 receptor subunit beta-2 (*il-12rβ2*), chemokine (C-X-C motif) receptor 2 (*cxcr2*), interleukin-17 receptor A-like (*il-17ra*), interlukin-8 (*il-8*), C-C motif chemokine 4-like (*cxcr4*), eosinophil peroxidase (*epo*), immunoglobulin tau heavy chain (*igt*).

**Table 3:** Effects of *C. vulgaris* on serum biochemical indices of *M. salmoides*.

Dietary *C. vulgaris* Levels (g/kg)	ALT (U/L)	AST (U/L)	ALB (g/L)	TP (g/L)
0	3.78 ± 0.67	7.75 ± 1.06	13.77 ± 0.58 ^a^	46.56 ± 1.14 ^a^
38	3.29 ± 0.75	10.71 ± 1.21	17.09 ± 0.42 ^b^	49.52 ± 0.70 ^ab^
76	3.63 ± 0.85	10.85 ± 2.22	17.54 ± 0.34 ^b^	51.62 ± 1.52 ^b^
114	2.79 ± 0.38	10.99 ± 1.87	15.60 ± 0.81 ^ab^	45.11 ± 0.97 ^a^
152	1.49 ± 0.27	8.84 ± 0.60	15.18 ± 0.67 ^ab^	44.78 ± 1.53 ^a^

Note: Alanine aminotransferase (ALT), aspartate aminotransferase (AST), albumin (ALB), and total protein (TP). The data were described in terms of mean ± standard error. Values with different alphabetical superscripts were significantly different (*p* < 0.05).

**Table 4:** Quality evaluation of sequencing data.

Sample	Total Reads	Valid Ratio (%)	Q30 (%)	GC Content (%)
CHL0-1	40,723,120	95.40%	94.74	47.26
CHL0-2	44,880,612	96.08%	95.01	47.33
CHL0-3	43,214,730	96.13%	94.88	47.26
CHL76-1	44,842,096	96.66%	95.08	49.08
CHL76-2	47,363,898	95.60%	94.19	47.35
CHL76-3	38,524,894	95.53%	93.62	47.43

**Table 5:** Information statistics of DEGs in the intestine of juvenile *M. salmoides* fed CHL76 diet compared to that fed CHL0 diet.

Signaling Pathway Name	Gene Name	*q* Value	Regulation	Log_2_FC
Apoptosis	*pro-cathepsin H*	0.000	up	2.46
	*caspase-3-like*	0.000	down	−1.62
	*caspase-7*	0.000	down	−1.80
	*cathepsin K-like*	0.000	up	9.66
	*ctsl1*	0.000	up	3.51
Cytokine–cytokine receptor interaction	*il-12rβ2*	0.000	down	−1.25
	*cxcr2*	0.001	down	−1.18
	*il-17ra*	0.048	down	−1.04
	*il-8*	0.032	down	−1.00
	*cxcr4*	0.002	down	−1.18
Tight junction	*claudin-4*	0.001	up	1.28
	*occludin b*	0.000	up	2.75
Phagosome	*igt*	0.000	up	5.94
	*epo*	0.000	up	1.53

Note: cathepsin L1 (*ctsl1*), interleukin-12 receptor subunit beta-2 (*il-12rβ2*), chemokine (C-X-C motif) receptor 2 (*cxcr2*), interleukin-17 receptor A-like (*il-17ra*), interlukin-8 (*il-8*), C-C motif chemokine 4-like (*cxcr4*), eosinophil peroxidase (*epo*), immunoglobulin tau heavy chain (*igt*).

## Data Availability

Data are contained within the article.
